# Validation of the Greek Version of the Colorado Learning Difficulties Questionnaire

**DOI:** 10.3390/children12121623

**Published:** 2025-11-29

**Authors:** Eleni Panagiota Fefe, Xanthi Tigani, Maria Michou, Vasiliki Efthymiou, Antonios I. Christou, Christina Darviri, Maria Charalampopoulou, Emmanouil Apostolakis, Stavroula Papadodima, Christina Kanaka-Gantenbein, Flora Bacopoulou

**Affiliations:** 1 Medical School, National and Kapodistrian University of Athens, 11527 Athens, Greece; 2 Clinic for Assessment of Adolescent Learning Difficulties, Center for Adolescent Medicine and UNESCO Chair in Adolescent Health Care, First Department of Pediatrics, Medical School, National and Kapodistrian University of Athens, Aghia Sophia Children’s Hospital, 11527 Athens, Greece; 3 Department of Special Education, University of Thessaly, 38221, Volos, Greece; 4 Breast Unit, First Department of Propaedeutic Surgery, Hippokratio Hospital, Medical School, National and Kapodistrian University of Athens, 11527 Athens, Greece; 5 First Center for Interdisciplinary Assessment, Counseling and Support (KE.D.A.S.Y.), 4th Health District, 10438 Athens, Greece; 6 Department of Forensic Medicine and Toxicology, Medical School, National and Kapodistrian University of Athens, 11527 Athens, Greece; 7 First Department of Pediatrics, Medical School, National and Kapodistrian University of Athens, Aghia Sophia Children’s Hospital, 11527 Athens, Greece

**Keywords:** Colorado Learning Difficulties Questionnaire, CLDQ, validation, learning difficulties, Greek, students, parents

## Abstract

**Highlights:**

**What are the main findings?**

- The Greek validation of the Colorado Learning Difficulties Questionnaire (CLDQ) demonstrated satisfactory reliability and strong construct validity among Greek parents and caregivers.
- Exploratory factor analyses supported a five-factor structure (Reading, Mathematics, Social Cognition, Social Anxiety, and Spatial Problems) consistent with the original version, with minor item variations.

**What is the implication of the main finding?**

- The Greek version of the CLDQ constitutes a valid and reliable instrument for screening learning difficulties in Greek children and adolescents.
- Its implementation can facilitate early detection and targeted interventions for students with learning challenges.

**Abstract:**

Background: Learning disabilities have a significant impact on children’s and adolescents’ academic progress; thus, early detection is of great importance. Parents play a key role, as they can provide valuable and unique information regarding their offspring’s learning challenges. The Colorado Learning Difficulties Questionnaire (CLDQ) is a quick and easy-to-use parent-report rating questionnaire evaluating specific areas of functioning that are most affected in children and adolescents with learning difficulties, such as reading, mathematics, social cognition, spatial abilities, and memory. This study aimed to examine the validity and reliability of the Greek version of the CLDQ. Methods: A cross-sectional design was employed in a sample of Greek parents and caregivers to test the Greek version of the CLDQ. The 20-item questionnaire was translated into Greek using the appropriate forward-backward translation. Results: The final sample consisted of 303 parents/caregivers of children and adolescents aged 11–17 years with learning difficulties. Analysis was consistent with the original scale and revealed five major factors labeled Reading, Mathematics, Social Cognition, Social Anxiety, and Spatial Problems. Contrary to the original version, two items loaded on two separate factors. Conclusions: The Greek version of the CLDQ demonstrated acceptable reliability and good validity. Consistent with the original version, exploratory factor analysis identified five distinct factors. These findings support the Greek version of the CLDQ as a valid and reliable tool for assessing and screening students with learning difficulties in Greece.

## 1. Introduction

Learning Disabilities (LD) are multifaceted and complex neurobiological disorders that can affect individuals across all cultures and continents. Often, they coexist with attention deficit hyperactivity disorder (ADHD), conduct disorder, anxiety disorders, and depression [1], while the global prevalence of LD is estimated to range between 5 and 15% [2]. The aforementioned disorders are characterized by difficulties in academic performance, including learning, behavioral, emotional, and non-verbal learning disabilities (NVLD), whereas the child’s physical development and overall intellectual ability remain unaffected.

LDs are usually the result of a combination of hereditary and environmental factors [3]. Hence, it is of great importance to recognize that when a child’s social environment is unfavorable and complex, it can negatively affect their mental health and normal development, both socially and academically [4]. Such circumstances can be aggravated by excessive parental demands, stress, insecurity, improper teaching, loss, and sadness [4]. The overall prevalence of learning disabilities varies across populations, depending on the diagnostic criteria applied in each culture, as well as on other factors like stigma and reduced access to health services [5]. Often, stress can cause a sudden onset of learning disabilities. Comorbidity is also often observed between learning disabilities and eating disorders, bullying, autism, anxiety, self-harm, gender dysphoria, and other related issues.

Research has shown that reading difficulties increase with age and school grade. During the pandemic, a noteworthy increase in the cases of depression and gradual absenteeism from school was reported in this specific population [6]. The inability to meet age-appropriate academic expectations may lead to a sense of reduced motivation and dissatisfaction. Frequently, this difficulty works in a chain reaction, resulting in parents and caregivers of children with LDs being emotionally affected, often experiencing intense confusion, stress, and even depression regarding their children’s prospects both professionally and socially.

Parents, in particular, have a crucial and supportive role as the primary caregivers for children with learning difficulties over many years. They are strongly involved in their children’s ongoing remedial education after school, often facing many difficulties in managing both their children’s learning needs and their own emotional and behavioral issues at the same time. Additionally, children with learning difficulties often express intense anger, and in cases such as ADHD, hyperactivity further complicates therapy and renders it even more difficult for their caregivers to effectively help them. Hence, parents are often trying to find techniques or educational programs that will help them develop their skills as parents-caregivers [7].

Most importantly, parents play a key role in early detection and accurate assessment of their children’s learning difficulties, providing valuable and unique information for clinicians, researchers, and professionals, that is most of the time unavailable through other sources and settings. Hence, many tools designed to screen for specific learning difficulties in children are developed to be completed by parents or their caregivers. Among existing parent report tools, such as the Achenbach System of Empirically Based Assessment (ASEBA) and the parent-report Pandemic Anxiety Scale (PAS-P), which provide a significant source of information [8], the Colorado Learning Difficulties Questionnaire (CLDQ) provides a concise alternative focused specifically on learning domains [9]. The CLDQ was designed by Dr. Erik G. Willcutt and his research team to evaluate specific areas of functioning that are most affected in children and adolescents with learning difficulties, such as reading, mathematics, social cognition, spatial abilities, and memory. It includes 20 items within five scales, including reading, social functioning, social anxiety, spatial ability, and mathematics. It is among the very few tools that are specifically designed to screen for learning disorders and associated developmental challenges, and hence it is extremely valuable. A comprehensive assessment and understanding are provided when the adult identifies aspects of the child’s behavior that the child may be unaware of or unable to perceive. Early identification using a simple and quick tool like the CLDQ, which incorporates an observational approach, is also helpful in evaluating both the child’s behavior and the parent–child relationship [10].

This questionnaire has been widely used in English-speaking populations, and it has been translated and validated in the Persian language [11]. This validation has shown adequate reliability and validity of the tool, as it effectively differentiates children with learning difficulties while also providing valuable data from parents or caregivers about their child’s learning challenges. The aim of this study was to translate and validate the Greek version of the CLDQ in a sample of Greek parents.

## 2. Materials and Methods

### 2.1. Translation

Following contact with the creator of the questionnaire, Dr. Erik G. Willcutt, the research team was granted permission to translate and validate the questionnaire into the Greek language. Translation of the CLDQ was carried out using the forward/backward translation method. The forward translation was conducted by two independent, experienced bilingual health professionals familiar with the terminology of the questionnaire. The Greek version was discussed in an expert panel, including the two translators, a physician with expertise in pediatrics and adolescent medicine and on learning difficulties, and a psychologist. The Greek version was translated by a third bilingual professional translator into the English language, who had no prior knowledge of the scale. The Greek version was pre-tested on a small sample of 47 individuals to identify any misconceptions or difficulties in the comprehension of the scale’s items and to undertake any appropriate adjustments and corrections to be undertaken. More specifically, questions 3–11, 13–17, and 19 were revised for syntax, and in some cases, alternative wording was used to express the same meaning. After appropriate adjustments, a second test including 71 individuals was conducted as a pilot, and the questionnaire was finalized since no difficulties were reported.

### 2.2. Participants and Procedure

The research was conducted from April 2024 to September 2024. The questionnaire was administered online via a secure Google Forms platform, as provided through the National and Kapodistrian University of Athens platform.

Eligible participants were parents/caregivers able to read and write in the Greek language who agreed to their children and adolescents being assessed for learning disabilities. Both groups—parents/caregivers of children with diagnosed learning difficulties and those without—were included in the sample. Parents/caregivers of children diagnosed with disorders (at certified diagnostic centers) that may affect mental or cognitive function, apart from those in the high-functioning autism spectrum, were excluded from the study. A sample of over 300 parents/caregivers was considered optimal, as it exceeds the recommended minimum ratios (5–10 participants per item) for exploratory factor analysis [12,13]. Some participants were excluded from the final sample due to missing values. Specifically, cases with incomplete responses on key variables were removed following a listwise deletion (complete-case) approach, based on the criterion that essential data required for the planned analyses had to be fully available. Listwise deletion is a commonly applied method when missingness affects core variables and is assumed to occur completely at random (MCAR) [14].

Participants were recruited from the Clinic for Assessment of Adolescent Learning Difficulties of the Center for Adolescent Medicine and UNESCO Chair in Adolescent Health Care, First Department of Pediatrics, Medical School, National and Kapodistrian University of Athens, Aghia Sophia Children’s Hospital; from the 1st KE.D.A.S.Y. (Center for Interdisciplinary Assessment, Counseling, and Support) of the 4th Health District of Athens; and from a community sample, specifically through groups of teachers, special educators from special schools, and those who provided parallel support to children/adolescents, with parents/guardians completing the university’s online Google Forms.

Informed consent was obtained from all participants who agreed to participate, and a link to the study’s questionnaires (Google Forms) was provided via the National and Kapodistrian University of Athens online platform.

### 2.3. Measurements

#### 2.3.1. Colorado Learning Difficulties Questionnaire (CLDQ)

The CLDQ is a tool designed by Dr. Erik G. Willcutt, Richard Boada, Margaret W. Riddle, Nomita Chhabildas, John C. DeFries, and Bruce F. Pennington to measure the concerns of parents of children and adolescents who experience learning disabilities and was designed to provide a brief and easy screening measure for learning difficulties. It is a parent-report rating scale to screen for learning disabilities in children and adolescents, consisting of 20 items and covering five dimensions, including (a) reading, (b) mathematics, (c) social cognition, (d) social anxiety, and (e) spatial difficulties. Parents are asked to respond to each item of the CLDQ on a 5-point Likert-type scale ranging from 1 to 5 (1 = never/not at all, 2 = rarely/a little, 3 = sometimes, 4 = frequently/quite a bit, 5 = always/a great deal). Higher scores demonstrate greater levels of perceived academic difficulty [9].

#### 2.3.2. Sociodemographic and Medical History Questionnaires

Initially, family medical history and demographic data were recorded regarding age, gender (male/female), educational level (primary/secondary/tertiary/post-tertiary), employment (public employee/private employee/freelancer/retired/student/unemployed), marital status (married/single/widowed/divorced), working hours (<4 h/6 h/8 h/>8 h/not working), satisfaction from family income (0–5), number of children each parent/caregiver has (1/2/3/>3/twins/triplets), birth order of the child facing learning difficulties or other disorders (1st/2nd/3rd/4th/5th), and type of learning and other disorders of the child.

### 2.4. Statistical Analyses

Descriptive analyses were used to calculate the medians and interquartile ranges (IQRs) for the quantitative variables and the absolute and relative frequencies (%) for the categorical variables. Exploratory factor analysis was used to extract the factors of the CLDQ. The sample adequacy was assessed using the Kaiser-Meyer-Olkin (KMO) measure and Bartlett’s test of sphericity, and the varimax rotation method was applied. Eigenvalues greater than 1 showed the number of extracted factors. All the items had loadings greater than 0.3. For internal consistency, Cronbach’s alpha coefficient was evaluated. Due to the non-normality of the quantitative variables, the non-parametric Mann–Whitney U and Kruskal–Wallis tests were used. The normality of variables was assessed using the Kolmogorov–Smirnov and Shapiro–Wilk tests, as well as through kurtosis and skewness. All statistical analyses were performed with SPSS for Windows statistical software (version 22.0), and the significance level was set at *p* = 0.05. All tests were two-tailed.

## 3. Results

A total of 321 parents/caregivers initially participated in the study; however, 18 participants were not included in the analysis due to completion errors and missing values. Data from 303 parents/caregivers (64% female) were retained after exclusion of incomplete cases, with a median age of 46 years. The mean time for completion of the CLDQ was 5 min. Participants’ main characteristics are presented in Table 1. Almost two-thirds of the participants had completed tertiary education (65.3%). Regarding their employment status, more than half were private employees (55.4%), and 61.7% had 8 h workdays. Most participants were married (77.9%) and had two children (53.8%); the second children demonstrated learning difficulties (48.6%) and were mainly male (64.5%). The median age of the children was 14.3 years; they were mainly of Greek nationality (95.4%), and almost all of the parents were their biological parents (99%) (Table 1).

Table 2 presents the types of learning disorders and comorbidities among 303 children and adolescents. ADHD was the most common condition, reported in 31.0% of the sample. Dyslexia also appeared frequently (25.4%), followed by dyscalculia (16.8%) and dysgraphia (16.2%). Anxiety disorders were present in 10.6% of participants, while learning disabilities, in general, were also identified in 10.6%. Less common conditions included autism (3.6%), undiagnosed difficulties (3.0%), tic disorders (0.7%), and dysorthography (0.7%). Additionally, 7.9% of the participants were classified under “other” diagnoses. It is worth noting that there were other types of disorders that adolescents exhibited, including emotional immaturity, left-handedness, and depression.

Table 3 presents the results of the exploratory factor analysis of the 20 factors of the CLDQ as well as the internal consistency test using Cronbach’s a.

The adequacy of the sample size was tested with the Kaiser–Meyer–Olkin (KMO) test, and the KMO coefficient was 0.846. Bartlett’s test of sphericity had *p* < 0.001. The exploratory factor analysis showed that five factors had an eigenvalue >1 and explained 78.3% of the total variance. The loadings of all items were >0.508. The value of the Cronbach’s alpha coefficient for the five factors ranged from 0.898 for the social cognition factor to 0.948 for the spatial factor, indicating that there is satisfactory internal consistency. Finally, all the correlations between the items (item–total correlations) were >0.540.

Table 4 presents the results of sociodemographic characteristics along with the learning difficulties measured by the CLDQ tool.

As shown in Table 4, children whose mothers participated in the study appear to have more difficulties in the Reading subscale and the Spatial subscale (*p*-value = 0.002 and *p*-value = 0.008, respectively), compared to children whose fathers participated in the study. Regarding the biological sex of the children, based on the parents’ responses, girls scored higher in the Reading and Spatial subscales (*p*-value = 0.034 and *p*-value = 0.021) and lower in the Social Cognition and Social Anxiety subscales (*p*-value < 0.001 for both subscales) than boys. Regarding the educational level of the parents, it appeared that children of parents with a lower educational level scored higher on the Reading and Mathematics subscales (*p*-value = 0.026, *p*-value = 0.005, respectively) while they scored higher in Social Anxiety *p*-value < 0.001), compared to children with parents with a higher educational level.

Regarding working hours, on the Reading subscale, children of parents who worked > 8 h showed higher scores than children of parents who worked ≤8 h (*p*-value = 0.002). Correspondingly, children of parents who did not work scored higher than children of parents who worked ≤8 h on the Reading subscale. Additionally, children of parents who worked ≤8 h scored higher on the Social Cognition subscale compared to children of parents who worked >8 h (*p*-value = 0.029). Children of parents who worked>8 h scored higher on the Spatial subscale compared to children of parents who worked ≤8 h (*p*-value = 0.003).

According to marital status, children of single parents scored higher on the Social Cognition subscale than children of widowed parents, children of single parents scored higher on the same subscale than children of married parents, and children of divorced parents scored higher on the Mathematics subscale than children of married parents (*p*-value = 0.002). Children of divorced parents scored higher on the Mathematics subscale than children of married parents (*p*-value = 0.036).

Next, parents who had one child scored higher on the Social Anxiety subscale than parents who had >3 children, as did parents who had one child compared to three, parents who had two children compared to three, and parents who had one child compared to two (*p*-value = <0.001).

The order of birth of the children who have learning difficulties or other disorders did not reveal any significant difference in CLDQ subscales.

## 4. Discussion

The present study presents findings regarding the reliability and validity of the Greek version of the CLDQ. Our analysis was based on data collected from 303 parents of children who wanted to be assessed for learning difficulties. The factor structure was determined by the eigenvalues (higher than 1) and led to the creation of five factors, as follows: (1) Reading: representing the method of evaluation and understanding of reading difficulties; (2) Social Anxiety which refers to concerns about social appearance and social isolation; (3) Social Cognition, that is related to social functioning, reduced social skills, and social rejection; (4) Spatial: referring to developmental behavior, spatial functioning, and coordination and (5) Mathematics: including engagement with mathematical concepts, problem-solving, and calculations. These five dimensions have been supported in previous studies by the original developers and by research in other languages.

Contrary to the original English questionnaire, our study has shown that item 16 (On arithmetic problems, has difficulty keeping the numbers in columns?) and item 17 (Drawings look immature for age?), which belong to the spatial factor, loaded into different factors in the Greek sample. More specifically, item 16 loads to the factor “Mathematics” while item 17 loads to the Social Cognition factor. This differentiation may stem from cultural and linguistic factors in the Greek context, particularly regarding the way arithmetic and spatial skills are taught in Greek education, where tasks involving mathematical organization are closely integrated into early mathematics instruction.

It is also noteworthy that although some studies indicate that girls may experience greater difficulties in reading compared to boys—a pattern also observed in our study— other studies have shown that girls perform better in reading than boys [15,16]. However, both genders with dyslexia have writing and reading problems, whilst the boys’ writing difficulties are “likely to be more severe” [17]. Gender “disparities” in reading do not always appear, but when they are observable, it is easy to conclude that “males do better in numeracy and females do better in reading” according to researchers [15]. Nevertheless, it is of utmost importance to screen for early reading problems in children and adolescents with dyslexia and provide both genders with the appropriate intervention.

Additionally, with regard to spatial functioning, parents in our sample reported that girls faced greater challenges in spatial functioning. This factor relates to spatial abilities, with adolescents who have difficulty understanding and expressing linguistic cues, as well as those who struggle with healthy developmental functioning in terms of thinking and movement, being more susceptible. Research indicates that girls generally experience more difficulty with spatial skills compared to boys. According to available neurobiological data, male individuals tend to perform better in spatial processing and perception.

With regard to Social Cognition and Social Anxiety, it has been shown that children with learning disabilities often express social maladjustment and lack of interpersonal relationships [18]. Our findings suggest that girls generally perform better in terms of social knowledge compared to boys and face fewer challenges related to social anxiety problems.

Referring to a multitude of studies, it can be established that boys may more often experience attention deficit hyperactivity disorder because they appear to be more vulnerable to it, in contrast to girls, who tend to experience depression and anxiety more often [19]. Girls seem to encounter internalizing problems, while boys encounter externalizing problems. According to data from further studies, it is confirmed that girls have a higher prevalence rate of internalizing problems, especially when it is also influenced by the social status quo [20].

Finally, with regard to the Mathematics subscale, our findings suggest that children whose parents had completed secondary school tended to experience more difficulty in mathematics compared to children whose parents had higher education. Furthermore, children of divorced or unmarried parents showed significantly lower mathematics skills. This may relate to socioeconomic status, which has a direct impact on children’s educational outcomes. Research suggests that factors such as parental divorce, family income, separation, age, physical health at birth, physical learning disabilities, students from minority groups, and other variables can negatively affect academic performance, particularly in children with learning disabilities [17,21].

There are a few limitations in our study. Research could incorporate test–retest analysis to further assess reliability. Moreover, the study presented several limitations: (a) a lack of comparison with other validated measures, (b) the absence of a clinical versus non-clinical group, and (c) potential selection bias. Participants were mainly residents of the capital of Greece, Athens, and not from all cities and districts of Greece.

## 5. Conclusions

The Greek version of the CLDQ demonstrated good psychometric properties, with adequate construct and criterion validity and internal consistency. Hence, the Greek version of CLDQ can be a valuable tool both for researchers on the subject and professionals who need to employ a quick, short, and reliable screening tool for early screening of learning difficulties in the Greek population.

## Figures and Tables

**Table 1:** Parent/caregiver sociodemographic characteristics (N = 303).

Variables	N (%) or Median (IQR)
Parent’s biological sex	
Female	194 (64.0)
Male	109 (36.0)
Parent/caregiver’s age (years) *	46.0 (5.0)
Education level	
Primary	1 (0.3)
Secondary	27.1)
Tertiary	198 (65.3)
Post-tertiary	22 (7.3)
Employment status	
Public employee	60 (19.8)
Private employee	168 (55.4)
Self-employed	53 (17.5)
Retired	4 (1.3)
Unemployed	18 (5.9)
Working hours	
<4 h	1 (0.3)
6 h	18 (5.9)
8 h	187 (61.7)
>8 h	76 (25.1)
Not working	21 (6.9)
Marital status	
Married	236 (77.9)
Single	8 (2.6)
Divorced	57 (18.8)
Widowed	2 (0.7)
Number of children	
1 child	82 (27.1)
2 children	163 (53.8)
3 children	53 (17.5)
>3 children	5 (1.7)
Which child, by birth order, experiences learning difficulties or other disorders?	
1st child	127 (42.9)
2nd child	144 (48.6)
3rd child	24 (8.1)
5th child	1 (0.3)
Child’s biological sex	
Female	107 (35.5)
Male	194 (64.5)
Child’s age (years) *	14.3 (2.4)
Child’s ethnicity/race	
Greek	289 (95.4)
Other	14 (4.6)
Relationship with the child	
Biological parent	300 (99.0)
Stepfather/Stepmother	3 (1.0)

Notes: Values refer to absolute (N) and relative frequencies (%) or * median and interquartile range (IQR).

**Table 2:** Learning disorders and co-morbidities of children and adolescents (N = 303).

Variables	N (%)
Dyslexia (yes)	77 (25.4)
Learning Disability (yes)	32 (10.6)
Dyscalculia (yes)	51 (16.8)
Dysgraphia (yes)	49 (16.2)
ADHD (yes)	94 (31.0)
Anxiety Disorder (yes)	32 (10.6)
Tic Disorder (yes)	2 (0.7)
Autism (yes)	11 (3.6)
Dysorthography (yes)	2 (0.7)
Other (yes)	24 (7.9)
Undiagnosed (yes)	9 (3.0)

Notes: Values refer to absolute (N) and relative frequencies (%).

**Table 3:** Exploratory factor analysis (EFA) rotated factor loadings for the 20 CLDQ items, item correlations, and Cronbach’s α coefficients (N = 303).

Items	Reading	Social Cognition	Mathematics	Social Anxiety	Spatial	Item Total Correlation
1. Has difficulty spelling?	0.553					0.686
2. Has difficulty learning letters?	0.743					0.758
3. Has difficulty learning phonics (sounding out words)?	0.837					0.820
4. Reads slowly?	0.935					0.862
5. Reads below grade or expectancy level?	0.934					0.875
6. Required extra help in school because of problems in reading or spelling?	0.664					0.761
7. Poor understanding of interpersonal space?		0.727				0.752
8. Has difficulty knowing how others react?		0.912				0.869
9. Has trouble understanding the feelings of others?		0.909				0.835
10. Makes comments that show a lack of understanding of social situations, such as inappropriate jokes or insensitive remarks?		0.818				0.761
17. Drawings look immature for age?		0.508				0.540
16. On arithmetic problems, has difficulty keeping the numbers in columns?			0.901			0.853
18. Worse at math than reading and spelling?			0.882			0.852
19. Makes careless errors in math, such as adding when the sign indicates subtraction?			0.887			0.860
20. Trouble learning new math concepts?			0.908			0.875
11. Has difficulty making or keeping friends?				0.802		0.856
12. Isolates self in social situations?				0.903		0.911
13. Feels anxious or out-of-place in new social situations?				0.855		0.887
14. Handwriting is spatially disorganized?					0.846	0.900
15. Papers look disorganized or messy?					0.882	0.900
% of Variance	20.576	17.113	16.607	12.485	11.516	
Eigenvalues	4.115	3.423	3.321	2.497	2.303	
Alpha of scale	0.921	0.898	0.941	0.944	0.948	

**Table 4:** Differences in sociodemographic characteristics with respect to learning difficulties (CLDQ, N = 303).

Study Measurements	Categories	Reading	Social Cognition	Mathematics	Social Anxiety	Spatial
Parent’s/Caregiver’s Biological Sex	Male	0.17 (1.83)	0.20 (0.80)	0.75 (2.00)	1.00 (1.67)	0.50 (2.00)
	Female	1.00 (1.83)	0.00 (0.60)	0.75 (1.25)	0.33 (1.33)	1.00 (2.50)
	*p*-value	**0.002**	0.332	0.698	0.130	**0.008**
Child’s Biological Sex	Boy	0.33 (2.00)	0.20 (0.80)	0.75 (1.50)	1.00 (2.00)	0.75 (2.00)
	Girl	1.00 (1.50)	0.00 (0.20)	1.00 (1.00)	0.00 (1.00)	1.00 (3.00)
	*p*-value	**0.034**	**<0.001**	0.627	**<0.001**	**0.021**
Education Level	Till Secondary	1.00 (1.83)	0.20 (0.60)	1.00 (2.25)	0.00 (1.00)	1.00 (3.00)
	Higher education	0.75 (1.92)	0.00 (0.80)	0.75 (1.00)	1.00 (2.00)	0.50 (2.00)
	*p*-value	**0.026**	0.360	**0.005**	**<0.001**	0.057
Working Hours	≤8 h	0.33 (1.67)	0.00 (0.60)	0.75 (1.50)	1.00 (2.00)	0.50 (2.00)
	>8 h	1.33 (2.00)	0.10 (0.80)	0.88 (1.00)	0.00 (1.00)	2.00 (3.00)
	Not Working	1.50 (1.67)	0.60 (1.40)	0.75 (1.75)	0.67 (1.67)	1.50 (1.50)
	*p*-value	**0.002**	0.053	0.806	**0.029**	**0.003**
Marital Status	Single	1.50 (1.75)	1.30 (1.80)	1.50 (2.38)	1.50 (1.00)	1.75 (1.00)
	Married	0.67 (1.83)	0.00 (0.60)	0.75 (1.25)	0.33 (1.67)	1.00 (2.00)
	Divorced	1.33 (2.17)	0.20 (1.00)	1.00 (1.25)	0.33 (1.33)	0.50 (3.00)
	Widowed	1.17 (1.33)	0.00 (0.00)	0.25 (0.50)	1.50 (0.33)	1.25 (1.50)
	*p*-value	0.341	**0.002**	**0.036**	0.058	0.686
Number of Children	1 child	1.00 (1.83)	0.20 (0.80)	0.75 (1.50)	1.00 (1.33)	0.75 (2.00)
	2 children	0.50(1.83)	0.00 (0.80)	0.75 (1.00)	0.33 (2.00)	0.50 (2.00)
	3 children	1.17 (2.00)	0.00 (0.80)	0.75 (1.50)	0.00 (1.00)	1.00 (2.00)
	>3 children	0.50 (1.00)	0.00 (0.20)	2.75 (2.00)	0.00 (0.00)	0.00 (1.50)
	*p*-value	0.368	0.740	0.411	**<0.001**	0.307
Which child in order of birth has learning difficulties or other disorders?	1st child	1.00 (1.67)	0.20 (0.80)	0.75 (1.25)	1.00 (1.67)	1.00 (2.50)
	≥2nd child	0.50 (2.00)	0.00 (0.60)	0.75 (1.25)	0.33 (1.67)	0.50 (2.00)
	*p*-value	0.067	0.447	0.896	0.053	0.068

Notes: Values refer to median and interquartile range (IQR). Values in bold indicate statistically significant differences.

## Data Availability

The data presented in this study are available on request from the corresponding author.

## Abbreviations

The following abbreviations are used in this manuscript:

LD	Learning Disabilities
ADHD	Attention Deficit Hyperactivity Disorder
NVLD	Non-Verbal Learning Disabilities
ASEBA	Achenbach System of Empirically Based Assessment
PAS-P	Parent-Report Pandemic Anxiety Scale
CLDQ	Colorado Learning Difficulties Questionnaire
EFA	Exploratory factor analysis
KMO	Kaiser–Meyer–Olkin
